# Phylogeographic Analysis of Soft-Rot-Causing *Pectobacterium* spp. Strains Obtained from Cabbage in Serbia

**DOI:** 10.3390/microorganisms11082122

**Published:** 2023-08-21

**Authors:** Aleksandra Jelušić, Marco Scortichini, Sanja Marković, Petar Mitrović, Renata Iličić, Slaviša Stanković, Tatjana Popović Milovanović

**Affiliations:** 1 Institute for Multidisciplinary Research, University of Belgrade, Kneza Višeslava 1, 11030 Belgrade, Serbia; jelusic.aleksandra@gmail.com (A.J.); sanja.markovic@imsi.rs (S.M.); 2 Council for Agronomical Research and Economics (CREA), Research Centre for Olive, Fruit and Citrus Crops, Via di Fioranello, 52, I-00134 Roma, Italy; marco.scortichini@crea.gov.it; 3 Institute for Field and Vegetable Crops—National Institute of the Republic of Serbia, Maksima Gorkog 30, 21000 Novi Sad, Serbia; petar.mitrovic@nsseme.com; 4 Faculty of Agriculture, University of Novi Sad, Trg Dositeja Obradovića 8, 21000 Novi Sad, Serbia; renatailicic@gmail.com; 5 Faculty of Biology, University of Belgrade, Studentski Trg 16, 11000 Belgrade, Serbia; slavisas@bio.bg.ac.rs; 6 Institute for Plant Protection and Environment, Teodora Drajzera 9, 11040 Belgrade, Serbia

**Keywords:** phylogeographic analysis, TCS haplotype network, *Pectobacterium*, cabbage

## Abstract

The aim of this study was to establish a link between genetic diversity and the geographic origin of *Pectobacterium* strains belonging to three species—*P. carotovorum*, *P. versatile*, and *P. odoriferum*—isolated from cabbage in Serbia by comparing their sequences with those of strains sourced from different hosts and countries in Europe, Asia, and North America. Phylogeographic relatedness was reconstructed using the Templeton, Crandall, and Sing’s (TCS) haplotype network based on concatenated sequences of the housekeeping genes *dnaX*, *icdA*, *mdh*, and *proA*, while pairwise genetic distances were computed by applying the p-distance model. The obtained TCS haplotype networks indicated the existence of high intra-species genetic diversity among strains of all three species, as reflected in the 0.2–2.3%, 0.2–2.5%, and 0.1–1.7% genetic distance ranges obtained for *P. carotovorum*, *P. versatile*, and *P. odoriferum*, respectively. Five new haplotypes (denoted as HPc1–HPc5) were detected among cabbage strains of *P. carotovorum*, while one new haplotype was identified for both *P. versatile* (HPv1) and *P. odoriferum* (HPo1). None of the TCS haplotype networks provided evidence of significant correlation between geographic origin and the determined haplotypes, i.e., the infection origin. However, as haplotype network results are affected by the availability of sequencing data in public databases for the used genes and the number of analyzed strains, these findings may also be influenced by small sample size.

## 1. Introduction

Plant pathogenic bacteria from the genus *Pectobacterium* (fam. *Pectobacteriaceae*) cause disease symptoms (e.g., soft rot, wilt, and blackleg) on a wide range of angiosperm plant species, including economically important crops (e.g., potato, tomato, cabbage) grown in geographically diverse regions (ranging from those with temperate to tropical climates) [1,2]. Their potential for long-distance dissemination is related to the ability to colonize host plants from various environmental sources, including soil, aerosols, irrigation water, groundwater, rainwater, non-host plants, and insects in the vicinity of arable land, as well as from some remote sources, such as winter mountain snow, waterfalls, rivers, seas, oceans, etc. [3]. Aside from its wide host range, the genus *Pectobacterium* is known for its high genetic heterogeneity, both within and between species [4]. While this aspect was previously insufficiently investigated, prompted by the development of new molecular tools and advanced techniques for assessing bacterial genetic diversity and phylogeny, the taxonomy of the genus *Pectobacterium* has recently received renewed research interest, resulting in the description of 20 species thus far [5,6,7].

Host adaptation/specialization, as well as horizontal gene transfer that enables *Pectobacterium* species to exploit distinct ecological niches and adapt to environmental changes, are considered the main drivers of their evolution [6,8]. Accordingly, comparison of their DNA sequences is the most reliable way to quantify genetic variations (e.g., single nucleotide polymorphism, haplotype structure, synonymous and non-synonymous changes, recombination events, etc.) within and between natural populations [9,10]. The findings yielded by the analysis and comparison of DNA sequences of individuals coexisting within the same population or those of different populations allow us to address additional questions regarding the ongoing microevolutionary processes related to population structure, gene flow, past demographic bottlenecks, and expansions, or geographical colonization events, etc. [11]. However, such investigations require assessment of many strains that originate from different countries and hosts. Given that changes in metabolic genes may be related to the adaptation of strains to specific environmental niches and host plants, typing and analysis of protein-coding loci (multilocus sequence typing and analysis, MLST/MLSA) can provide sufficient data for distinguishing closely related species [12,13]. Moreover, since the process required for obtaining strains from culture collections is often prohibitively expensive, and permits for some bacterial pathogens can be difficult to acquire, multilocus phylogenetic analysis can be a viable alternative, given that it exploits the information that can be derived from genomic sequences that are already deposited in the public databases [e.g., the National Center for Biotechnology Information (NCBI) database and the Plant Associated and Environmental Microbes Database (PAMDB)]. Its outcomes would improve the current understanding of phylogeographic and evolutionary patterns, which may provide insight into the transmission routes (i.e., whether outbreaks have a common source) as well as enable the reconstruction of the evolutionary history [14]. The Population Analysis with Reticulate Trees (PopART) software can use such data as input for the analysis of the available population genetic data and for constructing popular haplotype networks such as Templeton, Crandall, and Sing’s (TCS), minimum spanning networks (MSNs), and median-joining networks (MJNs), thus helping visualize intra-species genealogical relationships as well as advance our knowledge of biogeography and the history of populations [15]. The use of TCS haplotype networks for the determination of phylogeography of other soft-rot-causing bacteria (e.g., *P. brasiliense* and *Dickeya dianthicola*) was previously demonstrated in the work performed by Marković et al. [16] on strains isolated from potato in Serbia, based on concatenated sequences of four housekeeping genes (*acnA*, *icdA*, *gapA*, and *mdh*). Thus far, the TCS algorithm has been successfully applied for providing a phylogeographic insight into the population diversity of bacterial species belonging to different genera, such as *Xanthomonas* [17,18], *Pseudomonas* [19], *Agrobacterium* [20], *Ralstonia* [21], and *Clavibacter* [22].

In Serbia, different *Pectobacterium* spp.—*P. atrosepticum*, *P. brasiliense*, *P. carotovorum*, *P. odoriferum*, *P. punjabense*, *P. zantedeschiae,* and *P. versatile*—have been isolated from various hosts [13,16,23,24,25,26,27,28,29,30]. Three of these species (*P. carotovorum*, *P. odoriferum*, and *P. versatile*) were recently described as causal agents of soft rot in cabbage in this region [13]. To substantiate the findings presented in this unique report, in the present study, we aimed to conduct more extensive research on their genetic heterogeneity, as well as their spread routes and origin by performing phylogeographic analysis.

## 2. Materials and Methods

### 2.1. Sequences of the Pectobacterium spp. Strains Used for Phylogeographic Analysis

For the purpose of this investigation, the sequences of seven *Pectobacterium* spp. strains, namely *P. carotovorum* (Pc2321, Pc3821, Pc4821, Pc5421, and Pc8321), *P. odoriferum* (Po7521), and *P. versatile* (Pv6321), obtained from two cabbage hybrids [Cheers F1 (Takii Seed) and Hippo F1 (Sakata Seed)] grown in Futog (Vojvodina, Serbia) in 2021 were retrieved from the NCBI database. These seven Serbian cabbage strains were considered representative of each species based on their phenotypic and genotypic features determined previously by Jelušić et al. [13]. In order to reconstruct their phylogeographic relatedness, sequences of other 19 *Pectobacterium* spp. strains [*P. carotovorum* (ATCC 15713, 25.1, WPP14, BP201601.1, JR1.1, XP-13, and Pcc2520), *P. odoriferum* (BC S7, JK2.1, and CFBP 1878), and *P. versatile* (14A, 3-2, SCC1, F131, DSM 30169, MYP201603, SR1, SR12, and Pv1520)] obtained from different hosts (cabbage, carrot, Chinese cabbage, chicory, coleslaw, cucumber, kimchi cabbage, potato, and radish) and countries [Europe (Belarus, Denmark, Finland, France, Germany, Russia, and Serbia), Asia (China and Korea), and North America (USA)] were included in the analysis. The work performed as a part of this study is therefore a continuation of the previously conducted and published classical phylogenetic analysis based on the concatenated sequences of four housekeeping genes [*dnaX* (DNA polymerase III subunit tau), *icdA* (isocitrate dehydrogenase), *mdh* (malate dehydrogenase), and *proA* (gamma-glutamyl phosphate reductase)], for which the same tested and reference strains were used [13]. The maximal number of strains for comparative analysis was selected for each species in accordance with the availability of sequences for the four utilized housekeeping genes in the NCBI database. In the selection of genes for creating TCS haplotype networks, the appropriate sequence length and good discriminatory ability, as determined during previous work on molecular characterization of *Pectobacterium* spp. [13], served as the main criteria.

### 2.2. Phylogeographic Analysis

Phylogeographic relatedness of the Serbian cabbage strains belonging to three species—*P. carotovorum*, *P. odoriferum*, and *P. versatile*—was reconstructed using the TCS haplotype network [31]. TCS haplotype networks were generated for each species separately, based on partial concatenated sequences (1639 nt) of four housekeeping genes (*dnaX*, *icdA*, *mdh*, and *proA*), proposed by Sławiak et al. [32], Moleleki et al. [33], and Ma et al. [34]. Sequences were aligned using the ClustalW Multiple alignment function [35] of the BioEdit sequence alignment editor (v 7.2). Prior to the TCS network construction, DnaSP software v6 [9] was utilized to evaluate DNA polymorphism between the tested strains, as this approach allowed us to determine the maximal number of haplotypes present within tested strains isolated in different countries that served as inputs for the construction of TCS haplotype networks. As a part of the present study, the required TCS haplotype networks were generated using the TCS algorithm [36] implemented in the PopART v. 1.7 program [15]. Each circle on the TCS haplotype network represents one haplotype, while countries from which the reference strains used for comparative analysis originate are denoted by different colors. The number of hatch marks along the lines connecting haplotypes indicates the number of nucleotide differences (mutations) detected between those haplotypes. Further, the distribution of haplotypes by country/continent was graphically presented on the world map generated in the PopART program, whereby each differently colored circle signifies one haplotype.

Finally, pairwise genetic distances for the concatenated sequences of the seven tested and nineteen reference *Pectobacterium* spp. strains were computed in Mega software version 7.0, using the p-distance model/method. Genetic distances were computed for each species separately and standard errors (SE) were obtained by a bootstrap method with 1000 replicates.

## 3. Results and Discussion

### Phylogeographic Analysis

**Figure 1:**
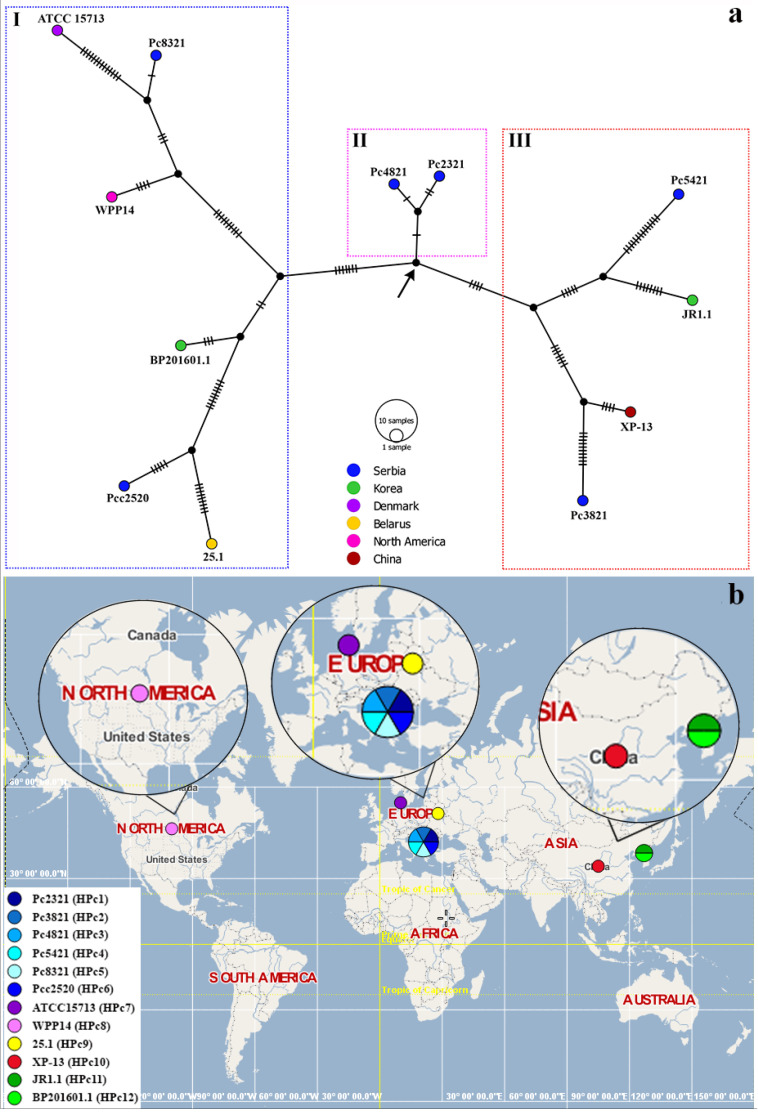
(**a**) Templeton, Crandall, and Sing’s (TCS) haplotype network showing the phylogeographic position of the five tested Serbian *P. carotovorum* strains (Pc2321, Pc3821, Pc4821, Pc5421, and Pc8321) and seven reference *P. carotovorum* strains (ATCC 15713, 25.1, WPP14, BP201601.1, JR1.1, XP-13, and Pcc2520). Different colors on the TCS network represent countries in which the tested/reference strains were isolated. The number of hatch marks reflects the number of nucleotide differences detected between haplotypes, while the arrow points to an ancestral genotype; (**b**) World map showing the distribution of the 12 detected haplotypes (HPc1–HPc12) of the tested and reference *P. carotovorum* strains by country.

The TCS haplotype network shown in Figure 1a was constructed based on the concatenated sequences of genes *dnaX*, *icdA*, *mdh*, and *proA* for the five tested Serbian *P. carotovorum* strains (Pc2321, Pc3821, Pc4821, Pc5421, and Pc8321) and seven reference *P. carotovorum* strains obtained from Belarus (25.1), Denmark (ATCC 15713), Serbia (Pcc2520), China (XP-13), Korea (BP201601.1 and JR1.1), and the USA (WPP14). As can be seen from the graph, each of the 12 tested *P. carotovorum* strains formed a single haplotype (designated as HPc1–HPc12). Based on their relatedness, haplotypes were divided into three major genetic clades/haplogroups (I–III) of the TCS haplotype network. Four of the six *P. carotovorum* strains/haplotypes placed within clade I were isolated in Europe (HPc5–HPc7, HPc9), while the remaining two were isolated in North America (HPc8) and Asia (HPc12), each exhibiting greater similarity with other clade I members relative to the strains placed into other two haplogroups (II and III). The centrally positioned haplogroup II consisted of only two Serbian *P. carotovorum* strains isolated from cabbage, Pc2321 (HPc1) and Pc4821 (HPc3), which are the most closely related to the ancestral vector (marked with an arrow), differing in three and two nucleotides, respectively. Finally, clade III included four *P. carotovorum* strains/haplotypes, two of which originated from Europe and were isolated from cabbage grown in Serbia [Pc3821 (HPc2) and Pc5421 (HPc4)], and two originated from Asia, and were isolated from radish [JR1.1 (HPc11)] in Korea and from potato in China [XP-13 (HPc10)]. As shown on the world map (Figure 1b) depicting all twelve haplotypes detected within the tested strains, six (HPc1–HPc6) were identified in Serbia, two (HPc11 and HPc12) in Korea, and one each in Denmark (HPc7), Belarus (HPc9), USA (HPc8), and China (HPc10).

The obtained TCS haplotype network clearly indicates the existence of a remarkable intra-species genetic heterogeneity within the tested and reference *P. carotovorum* strains. As specified above, while twelve haplotypes (HPc1–HPc12) were determined for strains originating from three continents (Europe, Asia, and North America) and from four hosts (cabbage, potato, cucumber, and radish), five of these haplotypes (HPc1–HPc5) were distinguished for the five Serbian cabbage strains (Pc2321, Pc3821, Pc4821, Pc5421, and Pc8321). The same five cabbage strains were separated into four clusters (I: Pc2321 and Pc4821, II: Pc3821, III: Pc5421, and IV: Pc8321) of the neighbor-joining phylogenetic tree previously generated by Jelušić et al. [13], thus confirming the existence of a complex population structure within the Serbian *P. carotovorum* strains isolated from this host. Population complexity is also reflected in the existence of four (HPc1–HPc4) *P. carotovorum* genotypes in a single cabbage field measuring only 0.5 hectares in size (field I—cabbage hybrid Cheers F1) [13]. However, based on the obtained TCS haplotype network, we cannot ascertain if there is a link between geographic origin and/or host of isolation and the determined haplotypes or make any assumptions regarding the infection origin. Nevertheless, it is important to emphasize that the reliability of phylogeny and haplotype networks is affected by the sample size, i.e., the number of strains included in the study, which depends on their availability in public databases as well as the choice of genes included in the research and their discriminatory ability. Despite the limitations imposed by the sample size, the obtained TCS haplotype network provides valuable information on the population structure of *P. carotovorum* in Serbia and other countries (Belarus, Denmark, China, Korea, and the USA) included in the analysis. Its benefit is further increased by the fact that, to the best of our knowledge, this is a pioneering study using the TCS haplotype network for exploring the phylogeography of *P. carotovorum*, as well as *P. versatile* and *P. odoriferum*. Thus far, only *P. brasiliense* strains from the *Pectobacterium* genus have been subjected to such analysis, which was based on a different combination of *acnA*, *gapA, icdA*, and *mdh* housekeeping genes [16]. The obtained findings indicate the existence of four haplotypes (PCB-1, PCB-2, PCB-3, and PCB-4) among twenty tested Serbian *P. brasiliense* strains from potato; however, no connection between the geographic origin and the genetic diversity was established [16]. The detection of a pronounced genetic diversity among *P. carotovorum* strains in this study is not surprising, given that it was previously confirmed by several authors. For instance, Gallelli et al. [37] indicated the existence of 14 haplotypes among 24 *P. carotovorum* strains isolated from artichoke in southern Italy (Sele valley, Campania), based on DNA profiling methods (repetitive-sequence-based PCR and M13-PCR). In the study conducted by Alvarado and colleagues, 39 tested *P. carotovorum* isolates collected from Chinese cabbage in north-eastern Brazil were shown to be polymorphic and were separated into 32 groups, also based on the repetitive-sequence-based PCR with REP-, ERIC-, and BOX-PCR primers [38]. According to Nabhan et al. [39], MLSA involving seven housekeeping genes (*acnA*, *gapA*, *proA*, *icdA*, *mtlD*, *mdh*, and *pgi*) enabled separation of sixty-three strains belonging to the *P. carotovorum* complex (including subspecies *carotovorum, odoriferum*, and *brasiliensis* based on earlier taxonomy), isolated from various hosts and countries, into five genetic clusters (PcI–PcV), three of which (PcI, PcII, and PcV) belonged to *P. carotovorum* subsp. *carotovorum* strains. However, these authors also failed to establish any correlation between the geographic origin and/or host affiliation and genotype [39].

The pairwise genetic distances between the tested and reference *P. carotovorum* strains calculated as a part of the present study shown in Table 1 provide support for the distribution of haplotypes on the TCS network. Genetic distances between the 12 compared *P. carotovorum* strains ranged from 0.2% (between Serbian strains Pc2321 and Pc4821 from cabbage) to 2.3% (between Serbian strains Pcc2520 and Pc3821 from potato and cabbage, respectively). Similar results were obtained for the five tested Serbian *P. carotovorum* strains from cabbage, as the estimated distance ranged from 0.2% (between strains Pc2321 and Pc4821) to 2.1% (between strains Pc5421 and Pc8321).

Similar findings were reported by Nabhan et al. [39], who calculated a 4.0% average genetic distance among *P. carotovorum* strains obtained from potato in Syria based on the sequences of seven housekeeping genes. Among all tested and reference *P. carotovorum* strains examined as part of the present study, Serbian strains from cabbage [Pc2321 (2.0%) and Pc4821 (2.1%)] were the most distant from the *P. carotovorum* type strain ATCC 15713 isolated from potato in Denmark, while the remaining three Serbian cabbage strains [Pc3821 (2.3%), Pc5421 (2.1%), and Pc8321 (1.6%)] were located furthest away from strain Pcc2520 isolated from potato in Serbia. These results confirm a highly complex and polymorphic *P. carotovorum* population structure in Serbia, irrespective of host type. The greatest similarity between strains Pc2321 (HPc1) and Pc4821 (HPc3) is also reflected in their grouping within the same cluster (haplogroup II) on the TCS haplotype network. Their percent identity with the strains deposited into the NCBI database ranged from 99.75% (*mdh*) to 100% (*dnaX*, *icdA*, or *proA*) depending on the used gene [13]. According to the previously performed phylogenetic analysis based on concatenated sequences [13] and the TCS network obtained in this study, cabbage strain Pc5421 (HPc4, haplogroup III) was the most closely related to the radish strain JR1.1 isolated from Korea (genetic distance 1.3%), while being the most divergent from the ancestral vector (differing in 23 nucleotides). The percent similarity of this strain with the strains deposited in the NCBI database ranged from 97.76% (*proA*) to 99.44% (*icdA*) [13]. Conversely, the strain Pc8321 (HPc5, haplogroup I) obtained from another cabbage hybrid (Hippo F1) and from another tested field (field II) was found to be the most closely related to the *P. carotovorum* type strain ATCC 15713 (genetic distance 0.9%). It also shared 97.91% (*proA*) to 100% (*dnaX*, *icdA*, and *mdh*) identity with the strains sourced from the NCBI database [13]. Although the *P. carotovorum* strains found in field II (1 ha in size) were genetically homogeneous in terms of the existence of only one haplotype (HPc5), while the remaining four (HPc1–HPc4) were detected on cabbage hybrid Cheers F1 (field I), it is important to emphasize that this cabbage hybrid harbored three pathogenic *Pectobacterium* spp. (*P. carotovorum*, *P. versatile*, and *P. odoriferum*) [13]. 

**Figure 2:**
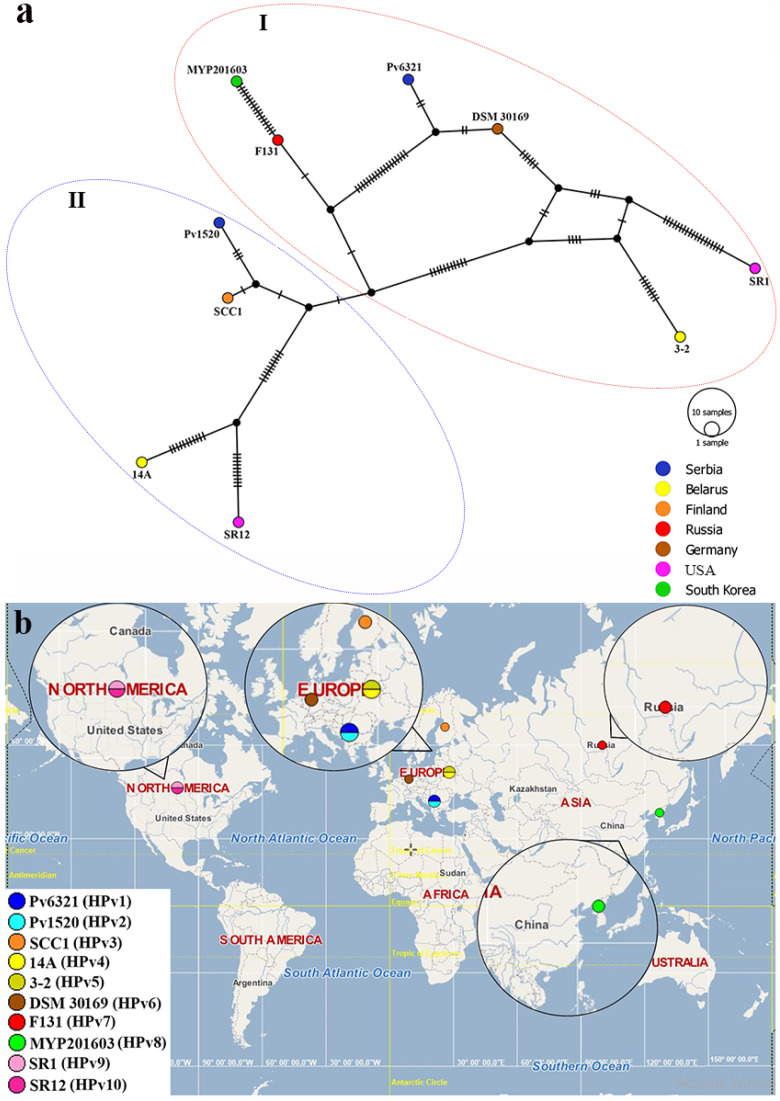
(**a**) Templeton, Crandall, and Sing’s (TCS) haplotype network showing phylogeographic position of one tested Serbian *P. versatile* (Pv6321) strain and nine reference *P. versatile* strains (14A, 3-2, SCC1, F131, DSM 30169, MYP201603, SR1, SR12, and Pv1520). Different colors on the TCS network represent countries in which the tested/reference strains were isolated. The number of hatch marks reflects the number of nucleotide differences detected between haplotypes; (**b**) World map showing the distribution of the 10 detected haplotypes (HPv1–HPc10) of the tested and reference *P. versatile* strains by country.

Figure 2a shows the TCS haplotype network generated for the 10 *P. versatile* strains [tested (Pv6321) and reference strains (14A, 3-2, SCC1, F131, DSM 30169, MYP201603, SR1, SR12, and Pv1520)]. These strains were separated into ten haplotypes (designated as HPv1–HPv10) that formed two clades/haplogroups (I and II) within the TCS network. The tested Serbian *P. versatile* strain Pv6321 (HPv1) from cabbage was placed within the haplogroup **I**, together with the five reference *P. versatile* strains, three of which were isolated from potato in Belarus (3-2, HPv5), Russia (F131, HPv7), and Korea (MYP201603, HPv8), one from cabbage in Germany (DSM 30169, HPv6), and one from carrot in the USA (SR1, HPv9). The *P. versatile* strain isolated from potato in Serbia (Pv1520, HPv2) was placed in haplogroup II with the strains isolated from potato in Finland (SCC1, HPv3) and Belarus (14A, HPv4), as well as from coleslaw in the USA (SR12, HPv10). Based on the presented world map (Figure 2b), two haplotypes were detected in Serbia (HPv1 and HPv2), Belarus (HPv4 and HPv5), and the USA (HPv9 and HPv10), while one haplotype was detected in Finland (HPv3), Germany (HPv6), Russia (HPv7), and Korea (HPv8).

Similar to the results reported for *P. carotovorum* strains, Serbian *P. versatile* cabbage strain Pv6321 formed a new haplotype (HPv1), which markedly differed from the haplotypes (HPv2–HPv10) detected for the reference *P. versatile* strains. As can be seen from Table 2, this strain was the most closely related to the German strain DSM 30169, which also originated from cabbage, differing in five nucleotides (p-distance 0.2%). With the exception of this relationship, the TCS network did not indicate any type of phylogeographic correlation between the tested and reference *P. versatile* strains. On the other hand, based on the calculated pairwise genetic distances, *P. versatile* strain Pv6321 was the most distant (differing in 39 nucleotides, p-distance 1.9%) from the strain MYP201603 isolated from potato in Korea (Table 2). 

The results yielded by examining the TCS haplotype network generated as a part of this study are in accordance with the phylogenetic tree previously obtained by Jelušić et al. [13]. The detection of 10 haplotypes for *P. versatile* strains with genetic distances ranging from 0.2% to 2.5% (Table 2) is indicative of a pronounced genetic polymorphism within the *P. versatile* species. In accordance with the number of strains from the three continents included in the comparison, seven *P. versatile* haplotypes were detected in Europe (HPv1–HPv7), two in the USA (HPv9 and HPv10), and one in Asia (HPv8). However, these results are completely dependent on the sample size, which is affected by (i) the number of publicly available strains (which can be limited due to the recent description of certain species), as well as (ii) the number and (iii) the combination of genes included in the comparison. Nonetheless, they concur with the report published by Ma and colleagues on a complex population structure of *P. versatile* in the northeastern United States based on the *dnaX* gene sequences [40]. The same authors indicated a much greater prevalence of *P. versatile* compared to other *Pectobacterium* spp. identified in this region, and ascribed this disparity to its better fitness and thus greater resilience to environmental conditions and/or cultivar characteristics. Given that *P. versatile* was recently described as a pathogen on cabbage in Serbia [13] and that no information regarding its geographic origin is currently available, it can be speculated that this species diverged over time from the dominant *P. carotovorum* populations through the accumulation of mutations as a result of the section pressure related to host, cultivar, or other pertinent factors. It is context, it is also worth noting that Park et al. [41] characterized the new *P. versatile* strain KNUB-02-21 on kimchi cabbage in Korea based on genes *dnaX*, *leuS*, and *recA*. These authors pointed to the existence of intra-species genetic heterogeneity among *P. versatile* strains (tested and reference), revealing three different genotypes among five compared strains. Based on three housekeeping genes (*dnaX*, *leuS*, and *gapA*), eight *P. versatile* strains isolated from cabbage (CaKh26, CaKh54, CaKh77, and CaKh83) and potato (PH35, PH47, PH62, and PH75) in Iran were divided into two clusters within the phylogenetic tree, each corresponding to one host [42].

**Figure 3:**
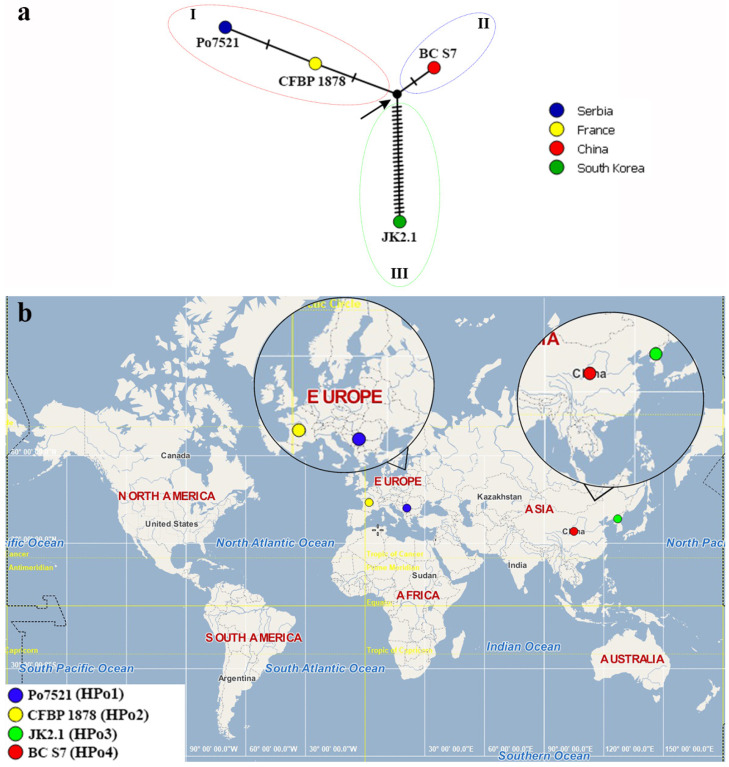
(**a**) Templeton, Crandall, and Sing’s (TCS) haplotype network showing the phylogeographic position of one tested Serbian *P. odoriferum* (Po7521) strain and three reference *P. odoriferum* strains (BC S7, JK2.1, and CFBP 1878). Different colors on the TCS network represent countries in which the tested/reference strains were isolated. The number of hatch marks denotes the number of nucleotide differences detected between haplotypes, while the arrow points to an ancestral genotype; (**b**) World map showing the distribution of the four detected haplotypes (HPo1–HPo4) of the tested and reference *P. odoriferum* strains by country.

As can be seen from Figure 3a, four detected haplotypes (designated as HPo1–HPo4) of the four tested (Po7521) and reference (BC S7, JK2.1, and CFBP 1878) *P. odoriferum* strains were divided within the TCS haplotype network into three clades/haplogroups (I–III) in relation to the ancestral vector (marked with an arrow), which occupied a central position. The Serbian *P. odoriferum* strain from cabbage Po7521 (HPo1) was placed within clade II, together with the type *P. odoriferum* strain CFBP 1878 (HPo2) isolated from chicory in France, from which it differed in one nucleotide only. Haplotypes of the reference *P. odoriferum* strains from Asia, isolated in Korea (JK2.1, HPo3) and China (BC S7, HPo4), were placed in clade II and III, respectively. Strain Po7521 differed from the ancestral vector in two nucleotides, while having only one different nucleotide relative to strains CFBP 1878 and BC S7. On the other hand, the strain JK2.1 differed from the ancestral vector in 26 nucleotides. In summary, as shown on the world map (Figure 3b), one haplotype was detected in each country—Serbia (HPo1), France (HPo2), Korea (HPo3), and China (HPo4).

Based on the TCS haplotype network constructed with tested and reference strains, a new haplotype of *P. odoriferum* (HPo1) was determined for the Serbian cabbage strain Po7521. The observed haplotype was the most similar to one of the *P. odoriferum* type strains [CFBP 1878 (HPo2)] isolated from chicory in France, differing in only one nucleotide (p-distance 0.1%, Table 3). On the other hand, it differed the most (in 28 nucleotides, Figure 3a) from the strain JK2.1 obtained from kimchi cabbage in Korea (p-distance 1.7%, Table 3). According to Jelušić et al. [13], the percent identity of Po7521 with the strains sourced from the NCBI database ranged from 99.57% (*proA*) to 100% (*icdA* and *mdh*), depending on the considered gene.

Once again, no correlation between geographic origin and/or the host of isolation could be established based on the constructed TCS network. These findings are supported by those reported by Oskiera et al., who determined the existence of intra-species genetic heterogeneity among *P. carotovorum* subsp. *odoriferum* strains isolated from cabbage and Chinese cabbage in Poland based on BOX- and ERIC-PCR, showing three and four distinct DNA fingerprinting patterns, respectively [43]. The genetic polymorphism among these strains was further confirmed based on the sequences of the 16S rRNA gene and five housekeeping genes (*gyrB*, *infB*, *rpoB*, *atpD*, and *rpoS*) [43].

Even though the TCS analysis performed in the present study focused on *P. odoriferum* and incorporated only a few strains, due to the recent description of this species and the scarcity of publicly available genomic data, the results reported in this work undoubtedly shed light on the issue of the phylogeography of the *Pectobacterium* genus. They also allow us to speculate that the two recently isolated species, *P. versatile* and *P. odoriferum*, could have been previously present on cabbage in Serbia, but due to their recent description and separation from the *P. carotovorum* group, they were not established in previous studies. We further posit that the observed intra-species genetic heterogeneity among cabbage strains might have arisen during the dynamic remodeling of genome content (gain, loss, duplication, and transfer of genes) which would have accelerated the evolution of the genus *Pectobacterium* [44].

## 4. Conclusions

TCS haplotype networks comprising sequences of *P. carotovorum, P. versatile*, and *P. odoriferum* strains isolated from cabbage in Serbia indicate high intra-species diversity among all three bacterial species. The results yielded by this study reveal five new haplotypes among *P. carotovorum* strains (HPc1–HPc5) and one new haplotype for both the *P. versatile* (HPv1) and *P. odoriferum* (HPo1) strains. These results provide further evidence of the usefulness of this approach in revealing closely related but different bacterial lineages that belong to the same species and that have been isolated from the same host plant in the same country. However, none of the TCS haplotype networks showed a correlation between geographic origin and the determined haplotypes among analyzed cabbage *Pectobacterium* strains.

## Figures and Tables

**Table 1:** Pairwise genetic distances (p-distance model/method) and standard errors (SE) between tested and reference *P. carotovorum* strains, calculated based on the partial concatenated sequences of genes *dnaX*, *icdA*, *mdh*, and *proA*.

*P. carotovorum* Strains	p-Distance/SE *											
	1	2	3	4	5	6	7	8	9	10	11	12
1. Pc2321		0.003	0.001	0.003	0.003	0.003	0.004	0.003	0.003	0.002	0.003	0.002
2. Pc3821	0.015		0.003	0.003	0.003	0.004	0.003	0.003	0.003	0.002	0.003	0.003
3. Pc4821	0.002	0.014		0.003	0.003	0.003	0.004	0.003	0.003	0.002	0.003	0.002
4. Pc5421	0.016	0.015	0.015		0.004	0.004	0.004	0.004	0.003	0.003	0.003	0.004
5. Pc8321	0.015	0.015	0.015	0.021		0.003	0.002	0.002	0.003	0.003	0.003	0.003
6. Pcc2520	0.016	0.023	0.016	0.021	0.016		0.004	0.003	0.002	0.003	0.003	0.002
7. ATCC 15713	0.021	0.020	0.020	0.020	0.009	0.020		0.003	0.003	0.003	0.003	0.003
8. WPP14	0.015	0.013	0.014	0.018	0.005	0.015	0.013		0.003	0.003	0.003	0.003
9. 25.1	0.019	0.019	0.018	0.018	0.013	0.009	0.018	0.014		0.003	0.003	0.003
10. XP-13	0.011	0.009	0.010	0.015	0.014	0.020	0.017	0.012	0.021		0.003	0.003
11. JR1.1	0.012	0.013	0.012	0.013	0.015	0.020	0.017	0.015	0.019	0.015		0.003
12. BP201601.1	0.009	0.016	0.009	0.020	0.012	0.010	0.015	0.012	0.012	0.016	0.015	

* Standard Error.

**Table 2:** Pairwise genetic distances (p-distance model/method) and standard errors (SE) between tested and reference *P. versatile* strains, calculated based on the partial concatenated sequences of genes *dnaX*, *icdA*, *mdh*, and *proA*.

*P. versatile* Strains	p-Distance/SE *									
	1	2	3	4	5	6	7	8	9	10
1. Pv6321		0.003	0.003	0.001	0.004	0.003	0.003	0.003	0.003	0.003
2. Pv1520	0.016		0.002	0.003	0.003	0.004	0.003	0.003	0.001	0.003
3. F131	0.013	0.004		0.003	0.003	0.004	0.003	0.003	0.001	0.003
4. DSM 30169	0.002	0.015	0.013		0.004	0.003	0.003	0.003	0.003	0.003
5. MYP201603	0.019	0.012	0.010	0.019		0.004	0.004	0.004	0.003	0.003
6. SR1	0.018	0.020	0.023	0.017	0.023		0.004	0.003	0.004	0.003
7. SR12	0.018	0.015	0.014	0.016	0.022	0.025		0.003	0.003	0.003
8. 14A	0.016	0.015	0.015	0.015	0.021	0.021	0.013		0.003	0.004
9. SCC1	0.015	0.002	0.003	0.013	0.013	0.022	0.013	0.014		0.003
10. 3-2	0.015	0.015	0.015	0.012	0.020	0.017	0.019	0.021	0.016	

* Standard Error.

**Table 3:** Pairwise genetic distances (p-distance model/method) and standard errors (SE) between tested and reference *P. odoriferum* strains, calculated based on the partial concatenated sequences of genes *dnaX*, *icdA*, *mdh*, and *proA*.

*P. odoriferum* Strains	p-Distance/SE *			
	1	2	3	4
1. Po7521		0.001	0.004	0.001
2. BC S7	0.002		0.003	0.001
3. JK2.1	0.017	0.016		0.003
4. CFBP1878	0.001	0.001	0.016	

* Standard Error.

## Data Availability

Not applicable.
